# Spontaneous Bladder Perforation in an Infant Neurogenic Bladder: Laparoscopic Management

**DOI:** 10.1155/2013/986362

**Published:** 2013-04-15

**Authors:** Daniel Cabezalí Barbancho, Felix Guerrero Ramos, Francisco López Vázquez, Adolfo Aransay Bramtot, Andrés Gómez Fraile

**Affiliations:** ^1^Pediatric Urology Division, University Hospital “12 de Octubre”, Complutense University, Avda Andalucia km 5,400, 28041 Madrid, Spain; ^2^Urology Department, University Hospital “12 de Octubre”, Complutense University, Avda Andalucia km 5,400, 28041 Madrid, Spain; ^3^Department of Pediatric Surgery, University Hospital “12 de Octubre”, Complutense University, Avda Andalucia km 5,400, 28041 Madrid, Spain

## Abstract

Spontaneous bladder perforation is an uncommon event in childhood. It is usually associated with bladder augmentation. We are presenting a case of bladder rupture in an infant with neurogenic bladder without prior bladder surgery. Three days after lipomyelomeningocele excision the patient showed signs and symptoms of acute abdomen. The ultrasound exploration revealed significant amount of intraperitoneal free fluid and therefore a laparoscopic exploration was performed. A posterior bladder rupture was diagnosed and repaired laparoscopically. Currently, being 3 years old, she keeps successfully dry with clean intermittent catheterization. Neurogenic bladder voiding function can change at any time of its evolution and lead to complications. Early diagnosis of spontaneous bladder rupture is of paramount importance, so it is essential to think about it in the differential diagnosis of acute abdomen.

## 1. **Introduction**

Spontaneous bladder rupture is uncommon. In children it is usually associated with bladder augmentation [1, 2]. The prognosis of this entity is subject to an early diagnosis and treatment. We present a case of bladder perforation in an infant without prior bladder surgery.

## 2. **Case Report**

A girl was diagnosed at birth with a lumbosacral lipomyelomeningocele with voiding habits appropriate for age. Urodynamic exploration performed at 10 months showed bladder capacity of 60 millilitres with normal intravesical pressure and no significant residual urinary volume. Lipomyelomeningocele was operated, with the girl being one year old. On the third postoperative day, she developed abdominal distension and discomfort accompanied by signs of acute abdomen. An ultrasound scan detected significant amount of intraperitoneal free fluid. With the presumptive diagnosis of urinary chemical peritonitis a diagnostic laparoscopy was performed.

**Figure 1:**
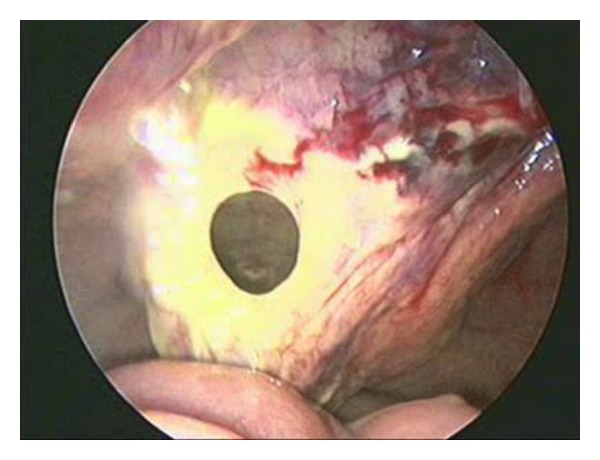
Laparoscopic imaging of bladder perforation.

The operation was carried out under general anesthesia with the patient in supine position. An infraumbilical incision was made down, and under direct vision a 5 mm port was placed into the peritoneal cavity. A 5 mm 30° telescope was used to visualize the operative field. Two additional 5 mm ports were placed under direct vision, one at the right lower iliac quadrant and the other in the left one. Necrosis of the posterior wall of the bladder with perforation was found. The perforation was identified, its edges resected, and the wall repaired in two layers with absorbable suture (Figure 1). A tube cystostomy was made and a urethral catheter placed for the postoperative recovery period. The patient clinically improved and no leak was demonstrated on cystography performed on the seventh postoperative day. We therefore decided urethral catheter removal and clean intermittent catheterization (CIC) every 3 hours maintaining the cystostomy closed. Currently, being 3 years old, she continues dry with CIC. Control ultrasound scan demonstrates both kidneys with normal size and morphology without pyelocaliceal ectasia; cystography shows normal bladder capacity with no vesicoureteral reflux. Urodynamic study shows atonic bladder with capacity and compliance poor for age.

## 3. **Discussion**

Spontaneous bladder rupture, defined as one that occurs in the absence of trauma or iatrogenic injury, is an extremely rare clinical entity in children [1–3]. Reviewing literature we found different etiologies for spontaneous bladder rupture [3–7]:

- obstruction caused by urine leaking, such as children with urethral valves or imperforate hymen,
- those caused by bladder wall weakness, as in cases of previous bladder surgery or diverticuli,
- low compliance after radiotherapy or in neurogenic bladders.

In our case, bladder perforation happened after removal of the urinary catheter in the immediate postoperative period after surgery of myelomeningocele. Presenting signs and symptoms depend on the age of the child and the severity of the rupture. They are nonspecific and include nausea, vomiting, fever, abdominal distension, acute abdomen, oliguria or anuria, and possible secondary sepsis. The medical record is the key to differentiate it from a urinary tract infection. Diagnosis is complex and associated with a high mortality rate [8–10]. Definitive diagnostic tests include computed tomography and retrograde cystography [11, 12]. In our patient the suspicion was raised after an ultrasound scan performed for the abdominal symptoms. The finding of free intraperitoneal fluid led us to carry out a laparoscopy exploration, which resulted in both diagnostic procedure and therapeutic procedure. Although our patient had no prior bladder dysfunction, it can be present throughout the development process of the child. 

## 4. **Conclusions**

All patients with myelomeningocele should be treated as potential cases of neurogenic bladder. Voiding function can change at any stage of development and lead to further complications. The laparoscopy can be used in both diagnostic procedure and therapeutic procedure in peritonitis of unknown origin. Early diagnosis of spontaneous bladder rupture, though infrequent, is of paramount importance, and therefore it is essential to include it in the differential diagnosis of acute abdomen in this kind of patients. 

## Abbreviations

CIC: Clean intermittent catheterization.
